# A Study of CC-Chemokine Receptor 5 (CCR5) Polymorphism on the Outcome of HCV Therapy in Egyptian Patients

**DOI:** 10.5812/hepatmon.13721

**Published:** 2013-12-19

**Authors:** Moataza H Omran, Mahmoud Khamis, Nada Nasr, Ahmed A Massoud, Samar S Youssef, Noha G. Bader El Din, Reham M Dawood, Khaled Atef, Rehab I Moustafa, Wael Nabil, Ashraf A Tabll, Mostafa K. El Awady

**Affiliations:** 1 Microbial Biotechnology Department, Genetic Engineering Division, National Research Centre, Cairo, Egypt; 2 Zoology Department, Faculty of Dentistery, Modern Science and Arts University (MSA), Cairo, Egypt; 3 Zoology Department, Faculty of Science, Tanta University, Cairo, Egypt

**Keywords:** Hepatitis C, Chemokines, Interferons, Host-Derived Cellular Factors

## Abstract

**Background:**

Chronic hepatitis C virus (HCV) infection is a globally serious public health issue.

**Objectives:**

In this study, we investigated CC chemokine receptor 5 (CCR5-59029) polymorphism which is considered an important component of the immune system in determining the outcome of HCV infection. Its critical role as a marker in response to interferon therapy of HCV infection is also investigated besides its effect on other clinical patient factors.

**Patients and Methods:**

This study was conducted on 82 Egyptian patients with chronic Hepatitis C Virus (HCV) infection who received PEG-INF + Ribavirin treatment for 48 weeks. The study was also conducted on 50 healthy controls (with negative results for HCV antibody and RNA PCR). Full history of patients in this study was recorded. Clinical and histological examinations, qualitative HCV nested RT-PCR, quantitative real –time PCR, and genotyping of HCV RNA genome were performed. CCR5-59029 polymorphism with nucleotide substitution from G to A was amplified. The amplicons were digested with restriction endonuclease Bsp 1286I, and produced RFLPs of the CCR5 genotypes were determined.

**Results:**

The present study showed a significant association between the functional SNP of CCR5 gene and the viral response to interferon in chronic HCV Egyptian patients. It was shown that the higher fibrosis stages (F2-F4) had significant association with nonresponse to treatment compared to the lower fibrosis stages (F0-F1) (95% confidence: 5.497 - 55.074, P = 0.0001). In addition, worse liver activity grade (A2-A3) had a very highly significant association with non-responder HCV patients compared to those with better liver activity grade (A1) (95% confidence: 2.242 - 20.974, P = 0.0007). Most importantly HCV patients with G allele had a high significant association with nonresponse to treatment, higher fibrosis stages and worse liver activity grades, while the A allele had a high significant association with sustained response, low fibrosis stages and relatively better liver activity grade (95% confidence: 3.347 - 15.036, P = 0.0001).

**Conclusions:**

SNPs within the CCR5 gene should be considered as an important factor used in combination with other host gene SNPs when developing a mathematical model for anticipating response to HCV therapy.

## 1. Background

Chronic hepatitis C virus (HCV) infection is an important public health issue. Globally, the estimated prevalence of hepatitis C virus infection is about 3%, and Egypt is considered the country with the highest HCV prevalence in the world (1). This mode of infection had now resulted in a high incidence of hepatic morbidity from the late complications of HCV infection, such as chronic hepatitis, cirrhosis, and hepatocellular carcinoma (1, 2). Interferon based therapies are the leading therapeutic regimens for the treatment of hepatitis C infection worldwide. Combination therapy of interferon and ribavirin or pegylated interferon had significant beneficial effects on virologic markers and histologic response. Despite the increased risk of hematological, dermatological and miscellaneous adverse events (3, 4). The chronic hepatitis C treatment efficacy is determined by the dynamic interaction of virus and host factors. Single nucleotide polymorphisms (SNPs), the most common forms of genetic variation could be useful as physical markers for comparative or evolutionary genomics studies as they could cause alterations in protein structure and function, leading to the development of disease or change in response to a drug (5, 6). In the recent studies of patients with chronic HCV, SNP analysis had been employed to predict both disease progression and therapeutic response as they include nucleotide polymorphisms of genes which function in the immune response such as interleukin-10, low molecular mass polypeptide 7 gene, and CC chemokine receptor 5 genes (7, 8). Chemokines are small heparin-binding proteins which direct the movement of mononuclear cells through the body to contribute to the development of an adaptive immune response and to the pathogenesis of inflammation. These Chemokines induce cell migration and activation by binding to specific cell-surface receptors on target cells, called chemokine receptors (9). HCV infection is controlled by an adequate specific T cell response, as they are able to migrate to the infected site to develop their effectors functions. Specific T cells fail to remove the virus in most patients as often a nonspecific T cell population is recruited to the infection site, and these cells are presumably responsible for the chronic damage (10). The attraction of T cells to the liver is controlled by chemokines, which are secreted by infected cells and interact with their receptors such as CCR5 expressed on the recruited T cells (9). Several chemokine and chemokine receptor polymorphisms have been associated with different HCV infection outcomes.

## 2. Objectives

Thus, the aim of our study was to investigate the association between (CCR5- 59029) gene polymorphism and response to interferon therapy in Egyptian patients with HCV infection and in addition to know the precise association between this polymorphism and other clinical patient factors within responder and non responder groups in our society.

## 3. Patients and Methods

### 3.1. Subjects

This study was conducted on 82 Egyptian patients (69 males and 13 females with mean age of 38 years old) with chronic HCV genotype 4 infection who received pegylated IFN + Ribavirin treatment for 48 weeks. Patients with chronic HCV infection were enrolled in the study. All patients had positive results for serum HCV-RNA and genotype 4a. Exclusion criteria included: 1) other causes of liver disease or infection by hepatitis B virus, 2) evidence of HCC at the time of inclusion as judged by negatives ultra sonographic findings, 3) uncontrolled diabetes mellitus, 4) history of long-term drug use, 5) alcohol abuse. Clinical history and laboratory examinations were recorded for all patients. Blood samples were taken before interferon therapy and after 48 weeks of treatment with a regular follow–up for 24 weeks after the end of treatment. Liver biopsy was performed for histological examination and assessment of the stages of fibrosis and activity. Liver fibrosis was scored into 5 stages from F0 to F4, and Liver activity grades were divided into 4 stages from A0 to A3. No side effects of IFN treatment were noted in any of the patients included in the study.

### 3.2. Controls

Fifty healthy subjects (n = 50) served as controls with no history of acute or chronic disease (negative results for HCV Ab and RNA with normal findings for liver enzymes), and absence of any other viruses, diseases or disorders. Written informed consent was obtained from both patients and controls and the study was approved by the ethical committee of the National Research Center. 

### 3.3. HCV RNA Tests

These include qualitative HCV nested RT-PCR, quantitative real –time PCR determination, and genotyping of HCV RNA genome. Methods used for these assays were previously described as follows; nested RT-PCR by amplifying the highly conserved HCV 5'-UTR region (10), Quantitative test of HCV RNA using (Cobas Amplicor, HCV monitor test Roche, The USA), HCV genotyping by nested PCR amplification of the HCV core gene using genotype specific primers (11).

### 3.4. DNA Extraction From Whole Blood Using Saltingout Technique (14).

#### 3.4.1. Molecular Typing of CCR5-59029 Polymorphism

**Figure 1.**
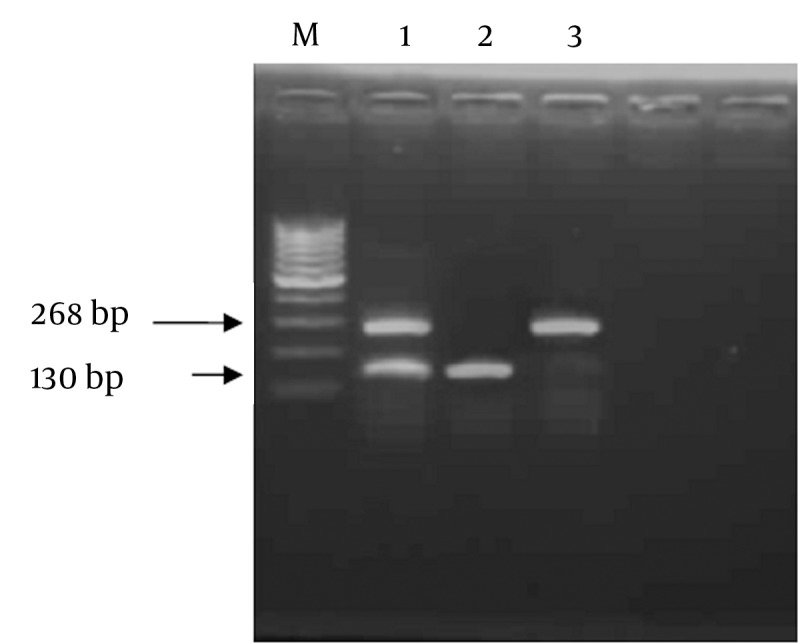
Agarose Gel Electrophoresis of Digested CCR5 -59029 Amplicons Purified genomic DNA from each patient was subjected to PCR amplification using specific primers of the CCR5 -59029, and the amplicon was digested using Bsp-1286 I, and the products were resolved on 2% agarose gel. Lane 1 represents heterozygous cut G/A; lane 2 shows homozygous cut G/G genotype, andlane 3 shows no cut A/A genotype and M indicated 100 bp marker.

Genomic variants of CCR5-59029 G/A were detected by RFLP technique through amplification by polymerase chain reaction ( PCR) using sequence specific primers as follows; Forward Primer (F): 5′- CCC GTG AGC CCA TAG TTA AAA CTC-3′, and Reverse Primer (R): 5′-TCA CAG GGC TTT TCA ACA GTA AGG-3′. Reaction mix (50 uL) contained 2 units Taq polymerase (finnzymes, Finland), 5 µL of 10X PCR buffer (supplied with the enzyme),0.2 mM dNTPs ( Promega, Madison WI, The USA), 1.5 mM MgCl2, 5 µM from each primer, 3 µL of DNA and DDW to 50 µL. Thermal cycling protocol comprised: initial denaturation at 94˚C for 5 minutes, followed by 35 cycles each including 94˚C for 30 sec, 53˚C for 45 sec, and 72˚C for 1 minute, and a final extension step at 72˚C for 10 minutes. Successfully amplified products of 268bp were digested with 10 units of Bsp-12861 restriction endonuclease (Amersham Pharmacia-Biotech, The UK) at 37°C overnight. Restricted fragments were resolved on 2% agarose gel electrophoresis parallel with a DNA size marker (Amersham Pharmacia- Biotech). The presence of GG genotype at the CCR5 -59029 was indicated by complete digestion of the 268 bp PCR product using Bsp-1286 I into two fragments of 138 bp and 130 bp (homozygous cut). While AA genotype was indicated by absence of Bsp-1286 I site where G to A polymorphism generated an indigestible sequence with a retained intact 268 bp PCR fragment (No-cut). In cases of heterozygosity (AG), the fragments of 268,130, and138 bp appeared on agarose gel electrophoresis (Figure 1). 

### 3.5. Statistical Analysis

The Statistical software program SaS was used for data analysis. Data was expressed as mean ± SD of percent (%) of each genotype (G/G, A/A or G/A) among subject population; whether, Sustained Virological Responder (SVR) or Non-Responder (NR). Comparison between mean values of different variables among SVR and NR patients was performed using unpaired student t test. Comparison between categorical data [No. (%)] was performed using Chi square test. Multinomial logistic regression with forward stepwise variable selection was used to identify predictors associated with SVR rates. Variables were described as odds ratio (OR) with 95% interval (CI). P values ≤ 0.05 were considered significant; highly significant if P < 0.01, and very highly significant if P < 0.001 (12, 13). 

## 4. Results

### 4.1. Clinical Data

The clinical and pathological parameters as age, ALT, alpha-fetoprotein, body weight, and viral load were studied with specific response pattern in a comparison performed between SVR [with sustained disappearance of HCV RNA levels in patient’s sera at least 6 months after the end of treatment] and Non-Response (NR) [where HCV RNA levels remain detectable in patients’ sera during or after the treatment] and outlined in Table 1. Alsoan association was shown between liver fibrosis and liver activity stages within SVR and NR patients in Table 1, where most of these factors showed higher values with nonresponders versus lower values in SVR patients. 

**Table 1.** The Demographic Data and Liver Function Tests in Egyptian Patients With Chronic HCV infection ^a^

Item	Non-responders, (n = 41)	Responders, (n = 41)	P value
**Age, Mean ± SD, y**	42.93 ± 1.45	33.63 ± 1.93	0.0002
**Gender**			0.432
Female, %	19.5	12.19	
Male, %	80.4	87.80	
**Body weight, Mean ± SD, kg**	81.83± 2.04	74.71± 2.78	0.421
**ALT ** ^**b**^ **, Mean ± SD, IU/L**	81.05± 11.11	46.17± 5.24	0.005
**AFP ** ^**b**^ **, Mean ± SD, level ng/ml**	22.35± 3.29	9.56± 3.54	0.0097
**Viral load, Mean ± SD, IU/ml**	70.27 × 104± 11.7x104	16.04 × 104± 7.6x104	0.0002
**Low Fibrosis, No. (%), (F0-F1)**	5 (12.20)	29 (70.73)	0.0003
**High Fibrosis, No. (%), (F2-F4)**	36 (87.80)	12 (29.27)	0.001
**Low Activity, No. (%), (A0-A1)**	21 (51.22)	36 (87.80)	0.0497
**High Activity, No. (%), (A2-A3)**	20 (48.78)	5 (12.20)	0.0056

^a^P values ≤ 0.05were considered significant. P value < 0.001= very highly significant. P value > 0.05 = not significant

^b^Abbreviations: ALT, Alanine aminotransferase; AFP, Alpha-fetoprotein.

### 4.2. CCR5-59029 Genotypes Within SVR and NR Patients

The present data showed that GG genotype was found to be significantly higher in NR group (n = 41) (80.49%) than SVR group (n = 41) (9.76%), while AG genotype was more statistically significant in SVR group (70.73%) than NR group (9.76%). Also AA genotype was detected more in SVR (19.51%) than NR group (9.76%) (Table 2). Analysis of the allele pool in the studied sample revealed that the frequency of allele A is more in SVR group (54.88%) than NR group (14.63%), while allele G is more in NR group (85.37%) than SVR group (45.12%) as shown in Table 2. 

**Table 2.** Statistical Analysis of the CCR5-59029 Genotypes and Alleles in NR and SVR Patients ^a^

	Non responders	Responders	Total	P value
Genotype				
**AA (n = 12)**	4 (9.76), (n = 41)	8 (19.51), (n = 41)	12 (14.63), (n = 82)	0.2577
**AG (n = 33)**	4 (9.76), (n = 41)	29 (70.73), (n = 41)	33 (40.24), (n = 82)	0.0002
**GG (n = 37)**	33 (80.49), (n = 41)	4 (9.76), (n = 41)	37 (45.12), (n = 82)	0.0001
**Allele**				
**A (n = 57)**	12 (14.63), (n = 82)	45 (54.88), (n = 82)	57 (34.75), (n = 164)	0.0001
**G (n = 107)**	70 (85.37) , (n = 82)	37 (45.12), (n = 82)	107 (65.24), (n = 164)	0.0017

^a^P values ≤ 0.05 were considered significant.P value < 0.001 = very highly significant. P value > 0.05 = not significant.

In studying the frequency of CCR5-59029 SNP in healthy controls versus infected patients, it was shown that the CCR5-59029 genotypes seem to be distributed equally with a very slight difference between controls and infected patients showing no specific association of certain allele with each group. AA was found in 12.5% of healthy controls versus 15% of patients, while GA was found in 37.5% of healthy controls versus 40% of patients; whereas, GG was found in 50% of controls versus 45% of patients similar to the earlier data (7)reporting the G allele frequency as 49.1% in healthy controls and 45.2% in patients with HCV infection.

### 4.3. CCR5-59029 Genotypes in Different Liver Activity Grades and Liver Fibrosis Stages

Patients with GG genotype had significantly higher fibrosis score (78.4%) than those with AG and AA genotypes (36.4% and 41.7%) (P = 0.0018) as shown in Table 3. Also patients with GG genotypes had significantly worse liver activity score (56.8%) than those with AG and AA genotypes (12.1% and 41.7 %) (P = 0.012) as shown in Table 3. 

**Table 3.** Frequencies of CCR5-59029 Genotypes and Alleles in Different Liver Activity Grades and Liver Fibrosis Stages ^a^

Factor	AA (n = 12), No. (%)	AG (n = 33), No. (%)	GG (n = 37), No. (%)	P value
**Fibrosis**				0.0018
F1	7 (58.33)	21(63.64)	8 (21.62)	
F2-F4	5 (41.67)	12(36.36)	29 (78.38)	
**Activity**				0.0123
A1	7 (58.33)	29(87.88)	16(43.24)	
A2-A3	5 (41.67)	4 (12.12)	21 (56.76)	

^a^P values ≤ 0.05 were considered significant. P value < 0.001 = very highly significant. P value > 0.05 = not significant.

### 4.4. Regression Coefficient Analysis of CCR5-59029 Genotypes with Different Host Factors in SVR and NR Patients

To study the effect of a single factor after controlling the effect of all other factors a stepwise logistic regression analysis was performed. Table 4 shows the significance of several host factors in determining the response to IFN therapy by multivariate statistical analysis of the different factors of fibrosis score, liver activity grades, AFP levels and alleles of CCR5-59029 G/A polymorphism in responders and non responders of HCV patients. HCV patients with higher fibrosis stages (F2-F4), worse liver activity (A2-A3), higher AFP levels > 5 ng/mL, and presence of G allele had a significantly higher risk of no response to IFN therapy. On the other hand those with lower fibrosis stages (F0-F1), better liver activity (A0-A1), lower AFP levels < 5ng/mL, and presence of A allele had a very highly significant association with the responders, (P = 0.0001, P = 0.0007, P = 0.0255, and P = 0.0001 respectively). 

**Table 4.** Multivariate Analysis of Different Clinical Factors and Alleles of CCR5- 59029 Polymorphism in HCV Patients ^a^

	Regression Coefficient	Standard Error	Odds ratio	95% Confidence Intervals	P value
**Fibrosis (F0-F1) vs. (F2-F4)**	2.8565	0.5879	17.400	5.497-55.074	0.0001
**Activity (A0-A1) vs. (A2-A3)**	1.9253	0.5704	6.857	2.242-20.974	0.0007
**AFP ( < 5 ng/ml vs. > 5 ng/ml)**	1.052	0.471	2.863	1.138-7.208	0.0255
**Alleles (A vs. G)**	1.9593	0.3832	7.094	3.347-15.036	0.0001

^a^P values ≤ 0.05 were considered significant. P value < 0.001 = very highly significant. P value > 0.05 = not significant.

## 5. Discussion

The progression of HCV infection is determined by the competence of the host’s innate and adaptive immune responses. HCV clearance is associated with vigorous HCV-specific CD4+ and CD8 + T cell responses (14). In contrast, lack of a sustained HCV-specific T cell response is associated with the development of persistent infection. Attraction of T cells to liver is controlled by chemokines, which are secreted by the infected cells and interact with their receptors such as CCR5 expressed on the recruited T cells. CCR5 is a CC chemokine receptor expressed on CD8+, and CD4+ T cells are responsible for recruitment of these crucial elements of immune response to the infection sites (9). 

The present work studied the CCR5- 59029 promoter as one of six chemokine system polymorphisms resulted in down regulation in CC-Chemokine Receptor 5 (CCR5) gene which is associated with resistance to interferon therapy in patients with HCV infection (15).It has been shown that demographic and clinical factors influence the likelihood of spontaneous HCV viral clearance. Age is one of the important parameters to treatment outcome. In our study, it was found that younger people respond better to current HCV treatments than older ones in agreement to other studies (8, 16, 17) as our data showed a significant difference of age between responders and non responders (P = 0.0002). In contrast, another study did not find any significant difference between the two studied groups (18). 

The high prevalence of HCV infection in males than females in our study could be due to this issue that males might be more exposed to the high risk factors of HCV transmission as surgeries, drugs, blood transfusion, Schistosomiasis infection, occupational exposure or might be the effect of different mechanisms of male hormones. However, further reports found no significant difference in gender distribution (8, 19). Also, the present work found no significant difference between body weights and response to IFN therapy similar to an earlier study (19). On the other hand an earlier report showed an association between patient’s weight and response to interferon therapy (20). 

In this study, the mean value of ALT in responders was about a half of the mean value of ALT in non responders, confirming the use of ALT activity as a marker of the efficacy of antiviral treatment in chronic hepatitis C (21-23). However, it was reported that response to IFN and ribavirin therapy in patients with chronic hepatitis C might not be associated with the levels of serum ALT (24). Our data showed that the mean value of viral load in non responders was three times more than responders revealing the importance of the baseline viral load as an active role in SVR rates (8, 23, 25). Moreover, the mean value of AFP in non responders group was twice as the responders group in accordance to the former results (26).

CCR5 is one of the specific receptors for pro-inflammatory chemokines as it is responsible for mononuclear cell accumulation in progressive liver injury and the proteins of its allelic variants have been shown to be important in the pathogenesis of viral infection, either by modulating virological response or by influencing the severity of liver injury. Furthermore, it was reported that chemokine polymorphisms play important roles in spontaneous clearance or persistence of HCV infection in Tunisian population (27).

In this work, the data revealed significant association between CCR5-59029 G/A polymorphism and response to interferon therapy in Egyptian patients with HCV infection of genotype 4. The study showed that patients harboring AG and AA genotypes tend to achieve significant sustained response to interferon therapy (87.9% and 66.6% respectively). Furthermore, the ratio of A allele distribution was found to be 54.88% in responders vs. 14.63% in non responders (P = 0.0001), suggesting that the A allele confers a better signaling towards viral clearance. On the other hand, a clear association exists between GG genotype and resistance to interferon therapy as the distribution of the G/G genotype was 89.19% in non responders vs. 10.81% in responders (P = 0.0001). Moreover, the ratio of G allele distribution was also found to be 85.37% in non responders vs. 45.12% in responders (P = 0.0017), and these data were also very highly significant. 

These findings are evidenced by earlier reports that the G allele is associated with down-regulation of CC chemokine receptor 5 which is in turn associated with resistance to interferon therapy in patients with HCV infection; whereas, the A allele results in up regulation of CC chemokine receptor 5 (15, 28). Similarly, other studies reported that the G to A single nucleotide polymorphism (SNP) of CCR5-59029 promoter region seems to be functional, enhancing decreased risk of some infections (28, 29). 

The present work also showed the association between CCR5-59029 G/A polymorphism with different fibrosis stages. It was found that the percentage of G/G genotype (78.38%) was highest in advanced fibrosis stage (F3-F4), while the percentage of G/A and A/A genotypes were higher at the low fibrosis stage (F1) (63.64% and 58.33% respectively).In addition, an association was found between CCR5-59029 G/A polymorphism and different liver activity grades. It was found that the percentage of G/G genotype was highest at severe liver activity grade (A2-A3) (56.76%), while, the percentage of G/A and A/A genotypes was higher at better liver activity grade (A1) (87.88% and 58.33 % respectively).

The current results show that the A allele gives positive response to interferon therapy in contrast to the G allele which is associated with a higher probability of nonresponse. Published evidence about alteration of CCR5 function by polymorphism is still not well investigated. The mechanism of viral elimination associated with chemokine receptors is still not clear and further researches are needed to study the influence of SNPs on CCR5 function together with SNPs of other host genes, to clarify the effect of phenotypic differences of CCR5 genotypes during IFN therapy. However, a similar study indicated that positive A allele effect may be due to the finding that A allele expresses more cell surface CCR5 in CD4 + cells than others (30). Meanwhile, Promrat et al. (28) presumed that GG genotype could relatively lead to the non function protein of CCR5, and might therefore be related to the sustained inflammation and progression of liver disease. This hypothesis was supported by the results of the present study as mean ALT, mean AFP levels were higher in GG genotype compared to AA and AG genotypes. Patients with chronic HCV infection with positive results for G allele had significantly higher fibrosis scores than those observed in patients without A allele (78.3% in GG vs. 41.7% in AA, P = 0.0018). Generally, those patients with GG genotype were more likely to have advanced grades of fibrosis. 

The results of our study can be explained by the fact that presence of G allele leads to down-regulation of CCR5 expression on T-cells which impedes the clearance of the virus in spite of therapy (31). On the opposite side, it was found that the frequency of CCR5 GG in Japanese patients of genotype 2 was higher in the sustained responders (29%) than non responders (11%). This conflict might be due to the host genome difference between Egyptian and Japanese populations (7). 

All our data were confirmed in a multivariate statistical analysis of the different prognostic factors in both SVR and NR patients including Fibrosis score, Histology Activity Index (HAI), Alpha-Fetoprotein (AFP) levels, and CCR5 alleles. These analyses revealed that the higher fibrosis stages (F2-F4), worse liver activity (A2-A3), AFP values > 5 ng/mL, and HCV patients with G allele had a very high significant association with nonresponse versus the lower fibrosis (F0-F1), better liver activity (A0-A1), AFP values < 5 ng/mL, and HCV patients with A allele which had a high significant association with the responders, while the G allele had a very highly significant association with the non responders. 

To sum up, the present study revealed clear evidence for the critical role of the CCR5 receptor and its legend in the regulation of immune response and its role in HCV infection. It was previously explained that infiltration of mononuclear inflammatory cells which is a central feature of hepatitis C virus (HCV) infection expresses high levels of the CCR5 receptor which differs among CCR5 59029 genotypes (29).

Moreover, in the current study, it had been shown that the CCR5-59029 promoter SNP is an independent and significant determinant of the outcome of IFN therapy. By taking together the present data and the former findings of other host genes effectiveness on the outcome of viral response to IFN based therapy; this could lead to valuable components of predictive mathematical model for selecting cohorts of ideal patients before starting the course of treatment.
